# Synthesis, Neuroprotective Effect and Physicochemical Studies of Novel Peptide and Nootropic Analogues of Alzheimer Disease Drug

**DOI:** 10.3390/ph15091108

**Published:** 2022-09-05

**Authors:** Radoslav Chayrov, Tatyana Volkova, German Perlovich, Li Zeng, Zhuorong Li, Martin Štícha, Rui Liu, Ivanka Stankova

**Affiliations:** 1 Department of Chemistry, Faculty of Mathematics & Natural Sciences, South-West University “Neofit Rilski”, 2700 Blagoevgrad, Bulgaria; 2 G.A. Krestov Institute of Solution Chemistry, Russian Academy of Sciences, 153045 Ivanovo, Russia; 3 Institute of Medicinal Biotechnology, Chinese Academy of Medical Sciences and Peking Union Medical College, Beijing 100050, China; 4 Faculty of Science, Charles University in Prague, 128 43 Prague, Czech Republic

**Keywords:** memantine, glycine, nootropics, neuroprotective action, solubility

## Abstract

Glutamate is an excitatory neurotransmitter in the nervous system. Excessive glutamate transmission can lead to increased calcium ion expression, related to increased neurotoxicity. Memantine is used for treating patients with Alzheimer’s disease (AD) due to its protective action on the neurons against toxicity caused by over activation of N-methyl-D-aspartate receptors. Nootropics, also called “smart drugs”, are used for the treatment of cognitive deficits. In this work, we evaluate the neuroprotective action of four memantine analogues of glycine derivatives, including glycyl-glycine, glycyl-glycyl-glycine, sarcosine, dimethylglycine and three conjugates with nootropics, modafinil, piracetam and picamilon. The new structural memantine derivatives improved cell viability against copper-induced neurotoxicity in APPswe cells and glutamate-induced neurotoxicity in SH-SY5Y cells. Among these novel compounds, modafinil-memantine, piracetam-memantine, sarcosine-memantine, dimethylglycine-memantine, and glycyl-glycine-memantine were demonstrated with good EC_50_ values of the protective effects on APPswe cells, accompanied with moderate amelioration from glutamate-induced neurotoxicity. In conclusion, our study demonstrated that novel structural derivatives of memantine might have the potential to develop promising lead compounds for the treatment of AD. The solubility of memantine analogues with nootropics and memantine analogues with glycine derivatives in buffer solutions at pH 2.0 and pH 7.4 simulating the biological media at 298.15 K was determined and the mutual influence of the structural fragments in the molecules on the solubility behavior was analyzed. The significative correlation equations relating the solubility and biological properties with the structural HYBOT (Hydrogen Bond Thermodynamics) descriptors were derived. These equations would greatly simplify the task of the directed design of the memantine analogues with improved solubility and enhanced bioavailability.

## 1. Introduction

**Figure 1:**
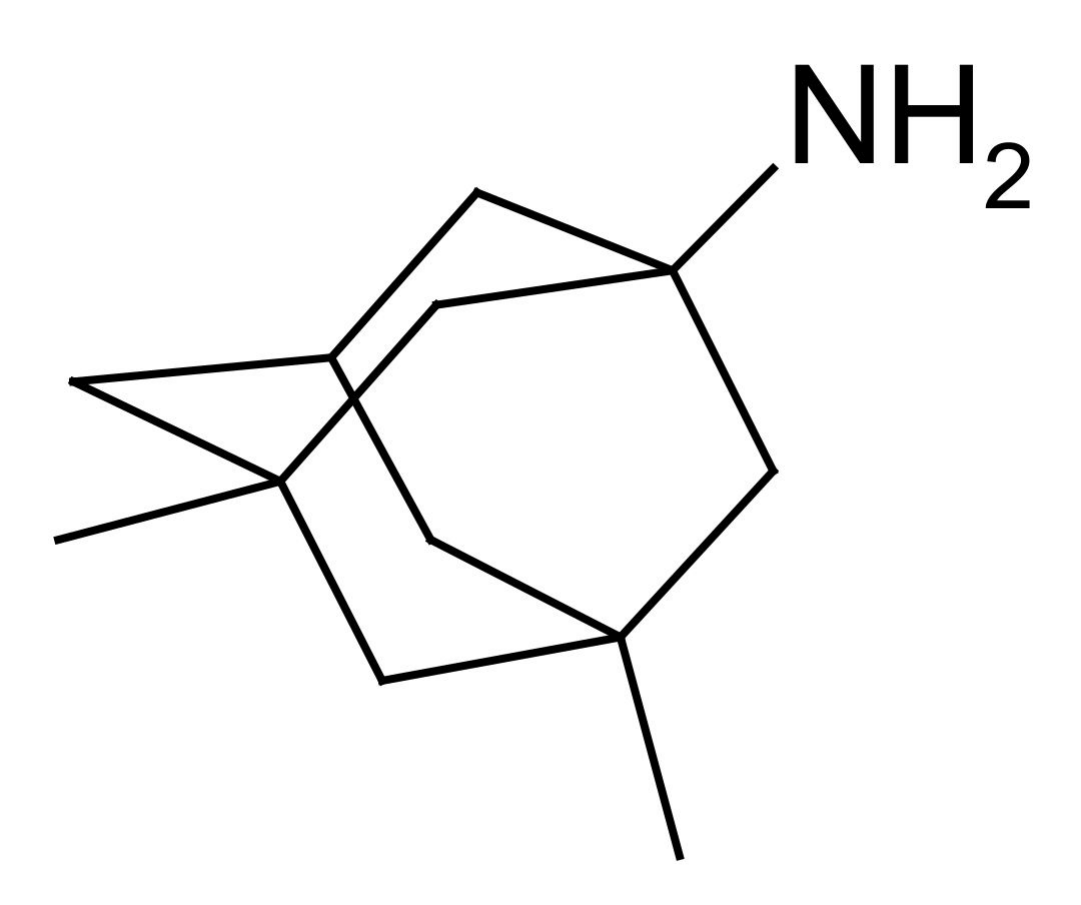
Chemical structure of memantine.

Dementia has been known and is a significant cause of disability, dependence and mortality. Memantine 1-amino-3,5-dimethyladamantan (Figure 1). It is used on patients with Alzheimer’s disease (AD). Progressive memory loss of and cognitive dysfunctions are the main symptoms. Currently this neurodegenerative disorder is irremediable. About 15 million people over the world are affected and are experiencing difficulties daily. The earlier symptoms come around the age of 65 and become more severe with age. Predominantly they are increasing from 0.5% at 65 to about 8% by the age of 85. Currently, AD is present in about 50–75% of all dementia patients [1,2,3].

Memantine action is based on the protection of neurons against toxicity caused by overactivation of N-methyl-D-aspartate receptors [4].

The concentrations of glutamate in the extracellular region should be low and is controlled by a multiple mechanism at the synapse. Disturbance to this system for regulation lead to harmful effects, for example, the excess releasing of glutamate. High levels of glutamate could cause increased excitability in post-synaptic neurons and excitotoxicity and cytotoxicity will probably appear. Glutamate-induced excitotoxicity in the hippocampus is related to less neuronal regeneration and dendritic branching, leading to weakened spatial learning. Any changes of glutamate uptake from the synapse are related to decreased sensitivity to reward, which is a symptom of depression. [5,6]. The main mechanism of excitotoxicity includes the excessive amounts of reactive oxygen radicals generated in overexcited neurons. On this basis, we can conclude that antioxidants could be effective in slowing down the development of neurological disorders by protecting the neurons from oxidative damage [7]. This state, called “glutamate neurotoxicity” or GNT, is explained by time-dependent cell injuries, which usually lead to cell death. In the biochemical mechanisms leading to the cell death the reactive oxygen species, known as ROS are hydroxyl radical (^•^OH), superoxide anion (^•^O^2−^), and hydrogen peroxide (H_2_O_2_). They are all generated in different cell parts due to various reactions. However, in the presence of some molecules which have an antioxidant activity the cells could prevent ROS damage. [8]. According to our earlier research, glycyl-glycine has demonstrated scavenging potential [9]. Dimethylglycine, known as dietary supplement DMG improves the immune system and decreases the oxidative damage by scavenging the excess free radicals [10,11,12]. Similar to betaine and choline, the DMG molecule increases antioxidant capacity because it is a building material for the glutathione synthesis [13]. Another study showed that DMG decreases oxidative damage and keeps the growth and health in researches with animals [14]. Abnormal high glutamate delivery could lead to increased calcium ion flow which is related to neurotoxicity. On the other hand, insufficient delivery could significantly change the information stream in neurons causing the symptoms similar to schizophrenia [1]. The amino acid and dietary supplement Sarcosine has the biological activity leading to the increase of normal functioning of the glutamatergic N-methyl-D-aspartate receptors (NMDAR). This action can be considered as a rational treatment for schizophrenia. There are a few studies which provides evidence for its efficiency [15,16].

Nootropics (modafinil, piracetam, picamilon etc.) help the process of learning and memory, attention and motivation. They facilitate recovery processes and improve the metabolism of nerve cells, protecting them from hypoxia. [17]. Over time these substances become dopaminergic and serotonergic drugs. However, we should pay attention to cholinergically active nootropics because of the crucial role of acetylcholine in learning and memory.

Modafinil, known as Provigil^®^ and Nuvigil^®^, is a widely preferred wake-promoting medication. It binds to the cell-membrane dopamine (DA) transporter competitively and is dependent on dopaminergic and adrenergic signaling for its wake-promoting action [18,19]. Additionally, Modafinil mediates the restoration of aerobic metabolism and hyper-glycolysis suppression, thereby resulting in an increase in pyruvate dehydrogenase and a decrease in lactate dehydrogenase activity, respectively, which ultimately reduced oxidative reperfusion injury [20].

The neuroprotective effect of piracetam has been well-known for a long time [21,22,23]. Piracetam has an influence on the excitatory neurotransmitters and inhibitors in the brain. It has been suggested that it has effects on increasing the availability of oxygen and permeability of the mitochondrial cell membrane in the Krebs cycle medium stages [24,25]. It has been reported that there are good effects of piracetam in the prevention of cognitive dysfunction related to the memory that usually occur after surgery under anesthesia [17].

Picamilon is a medication with proven cerebrovascular activity. From a chemical point of view, it is a nicotinoyl-gamma-aminobutyric acid and it is successfully used in neurological practice. Picamilon also exhibits antiplatelet activity [26,27,28].

Although there are various risk factors besides a genetic predisposition that are associated with the pathogenesis of such as oxidative stress, the formation of β-amyloid aggregates (Aβ), disorders in tau-protein, lowering of acetylcholine levels, dyshomeostasis of biometals, etc., the etiology of AD still remains a riddle. Acting as an open-channel blocker, the anti-AD drug memantine preferentially targets NMDAR overactivation, which has been proposed to trigger neurotoxic events mediated by amyloid β peptide (Aβ) and oxidative stress [29]. In this sense, the classic “drug-discovery” approach, based on the “one molecule-one target” paradigm, turned out to be ineffective. Based on the hypothesis that Alzheimer’s disease is a multifactorial disease, in the last 10 years there has been a strong interest in developing the targeted therapy to affect different targets in AD. Currently, of the few accessible symptomatic therapies for AD, memantine is the only N-methyl-d-aspartate receptor (NMDAR) blocker. Turcu et al. explores a series of memantine analogs featuring a benzohomoadamantane scaffold. Most of the newly synthesized compounds block NMDARs in the micromolar range [30]. The aim of the present study is the design of hybrid molecules, including two pharmacophoric moieties—memantine (NMDA antagonist) and nootropics. The choice of this approach is that the NMDA antagonist can possibly stop or slow neurodegeneration, while the nootropics can improve memory and cognitive abilities by stimulating the surviving neurons.

Along with the tests on the biological activity and receptor affinity the investigations aimed at the characterization of the physicochemical properties of drugs and newly synthesized biologically active substances are highly demanded on the early stages of development [31]. This comes from the need for acceleration of the rational selection of the compounds with the best characteristics and, as follows, shortening the time period to the pharmaceutical market. Aqueous solubility and permeability across the biological membranes are the main properties for study in the case of new drug candidates. Moreover, since the vast array of the drugs exists in different ionization state according to the pH of the respective medium, the solubility should be estimated in the solutions with different acidity. Another important issue in the rational drug design is the creation of the approaches to the improvement of the physicochemical properties which is often possible if the structure-property correlations are disclosed. Along with various methods for increasing the solubility of the substances, the structural modification of molecules is of great interest to specialists. This is due to the fact that the solubility of organic compounds directly depends on the features of their structure. Changing the structural fragments and introducing various substituents lead to the structural and stereochemical changes in the molecule. The latter, in turn, affect the physicochemical properties, including the solubility. Since the hydrogen bonds are critical in the most of chemical and biological processes, it seems reasonable to predict the solubility using the correlations based on the hydrogen bonding physicochemical descriptors (HYBOT). Taking into consideration the objects of the present study and the structural analogues—the memantine derivatives reported previously [32], we tried to disclose the correlations of the biological activity and solubility with the structure of the compounds.

## 2. Results and Discussion

Herein we report on a design, synthesis and investigate the neuroprotective activity of newly memantine derivatives, QSAR and solubility study experiments.

### 2.1. Chemistry

**Scheme 1:**
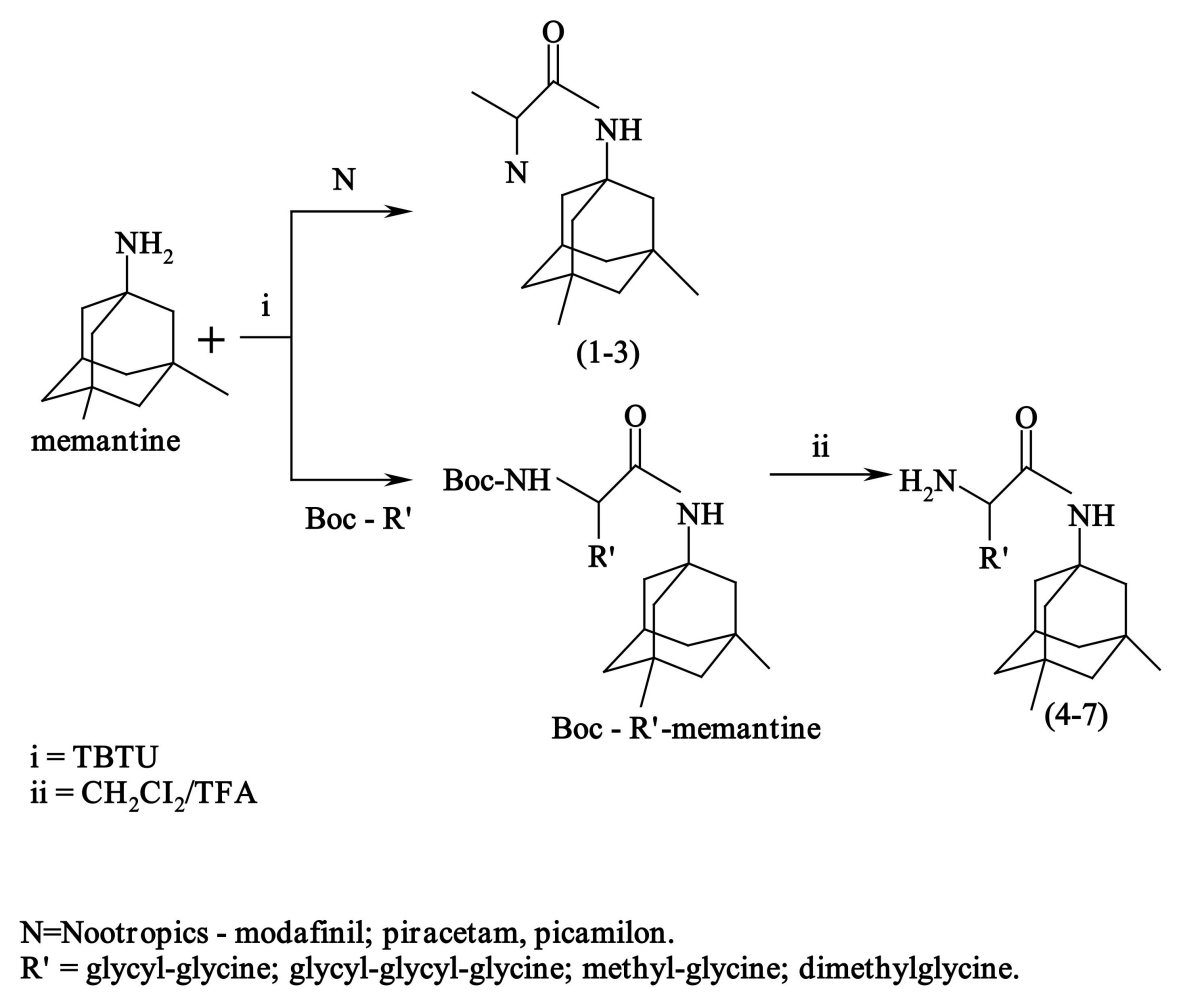
Synthesis of nootropics and glycine derivatives of memantine.

Three hybrid memantine molecules with nootropics—modafinil, piracetam and picamilon and four memantine analogues with glycine—glycyl-glycine, glycyl-glycyl-glycine, methyl-glycine and dimethylglycine (Scheme 1) were formed using the TBTU coupling reagent [33]. The *N*-Boc protecting groups of glycine analogues were removed in CH_2_Cl_2_/TFA at 0 °C and the final TFA salt was removed with aqueous ammonia.

**Figure 2:**
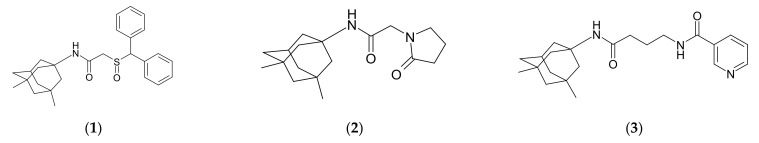

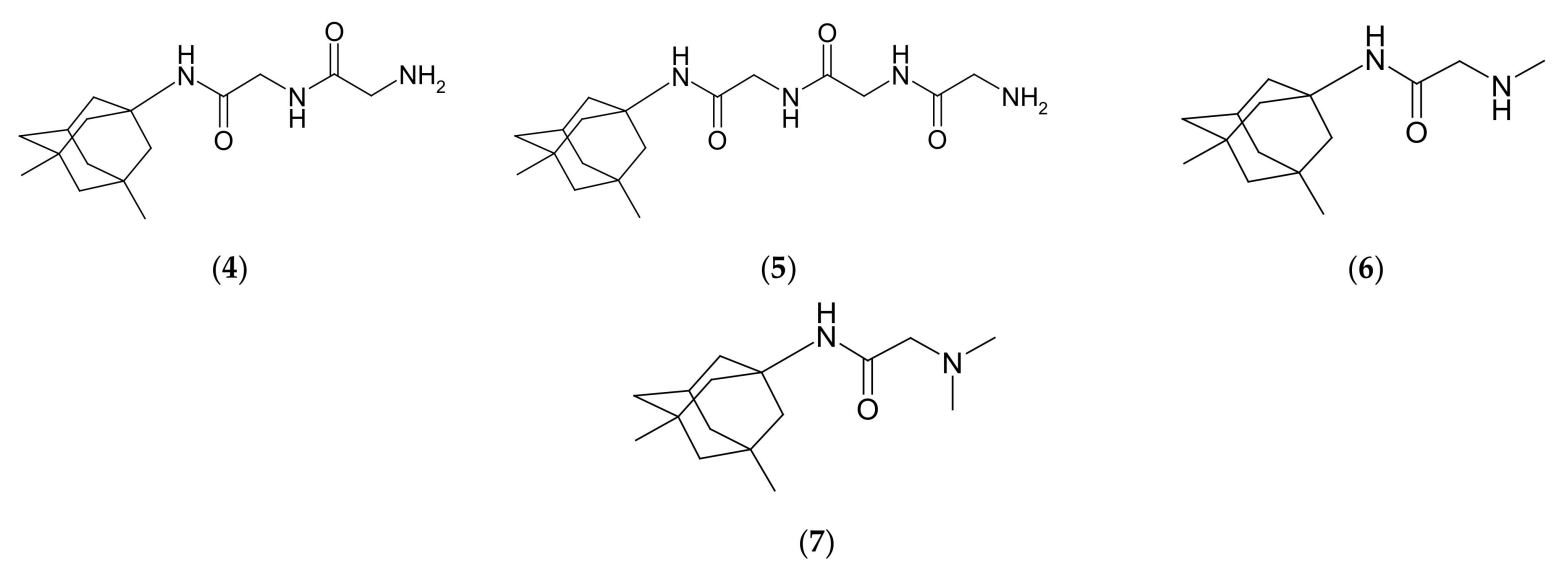
Chemical structures of the studied compounds. modafinil-memantine. (**1**); piracetam-memantine (**2**); picamilon-memantine (**3**); glycyl-glycyl-memantine (**4**); glycyl-glycyl-glycyl-memantine (**5**); sarcosine-memantine (**6**); dimethylglycyl-memantine (**7**).

The structures of the synthesized memantine analogues are shown in Figure 2.

### 2.2. Novel Structural Derivatives of Memantine Improve Cell Viability against Copper-Induced Neurotoxicity in APPswe Cells

**Figure 3:**
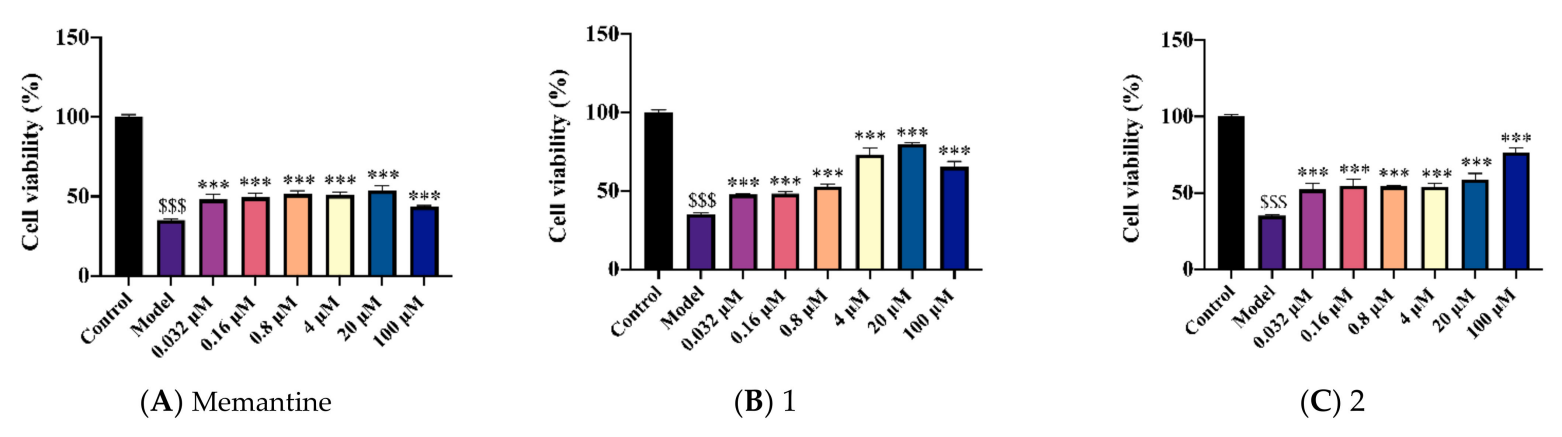

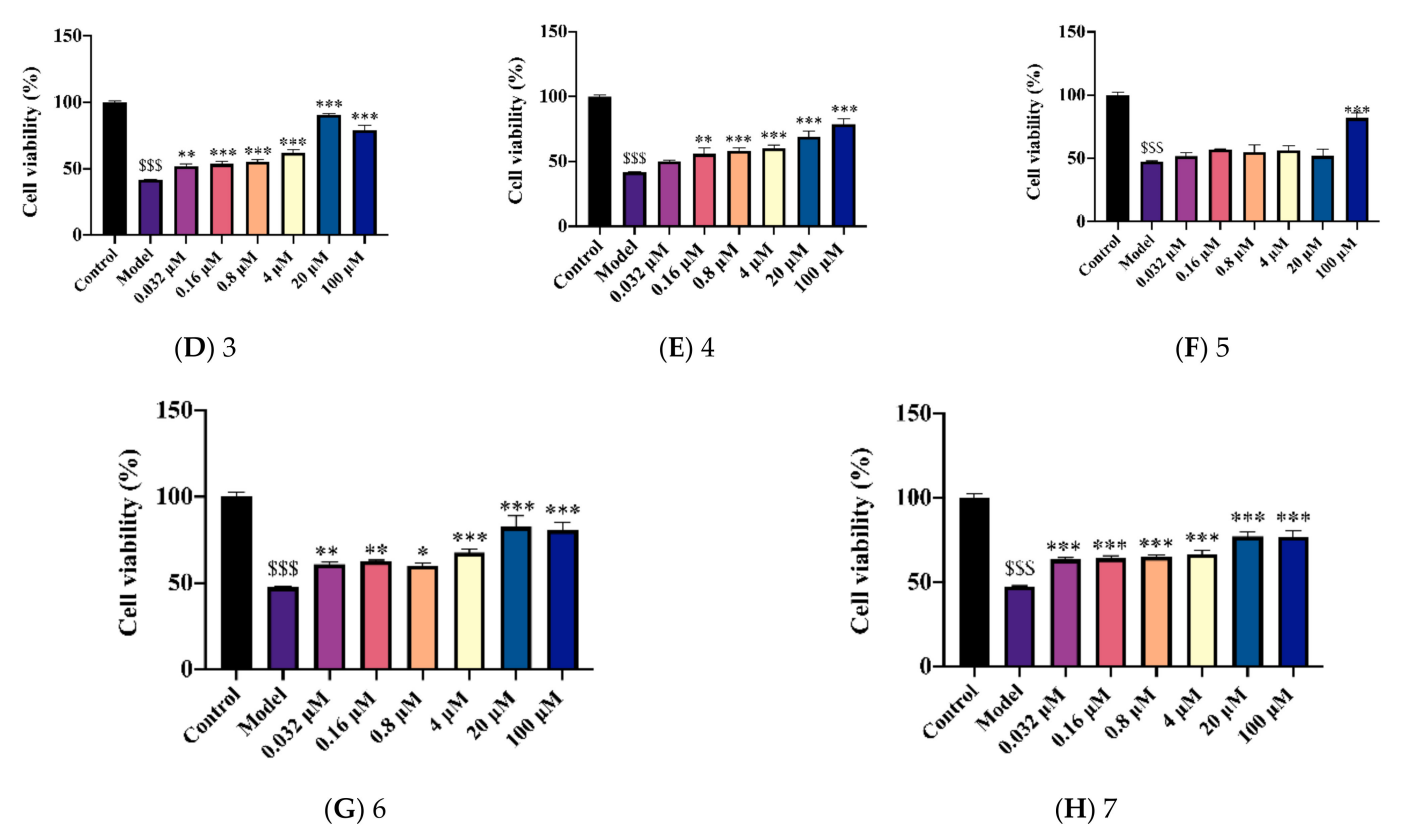
Cell viability of copper-injured APPswe cells treated with novel structural derivatives of memantine. (**A**–**H**) Cell viability of memantine (as a positive reference drug) (**A**) and memantine derivatives, including 1 (**B**) modafinil-memantine, 2 (**C**) piracetam-memantine, 3 (**D**) picamilon-memantine, 4 (**E**) glycyl-glycine-memantine, 5 (**F**) glycyl-glycyl-glycine-memantine, 6 (**G**) sarcosine-memantine, 7 (**H**) dimethylglycine-memantine, using MTS assay. Data are expressed as mean ± SEM, *n* = 3. ^$$$^ *p* < 0.001 vs. the control group; * *p* < 0.05, ** *p* < 0.01, *** *p* < 0.001 vs. the model.

**Figure 4:**
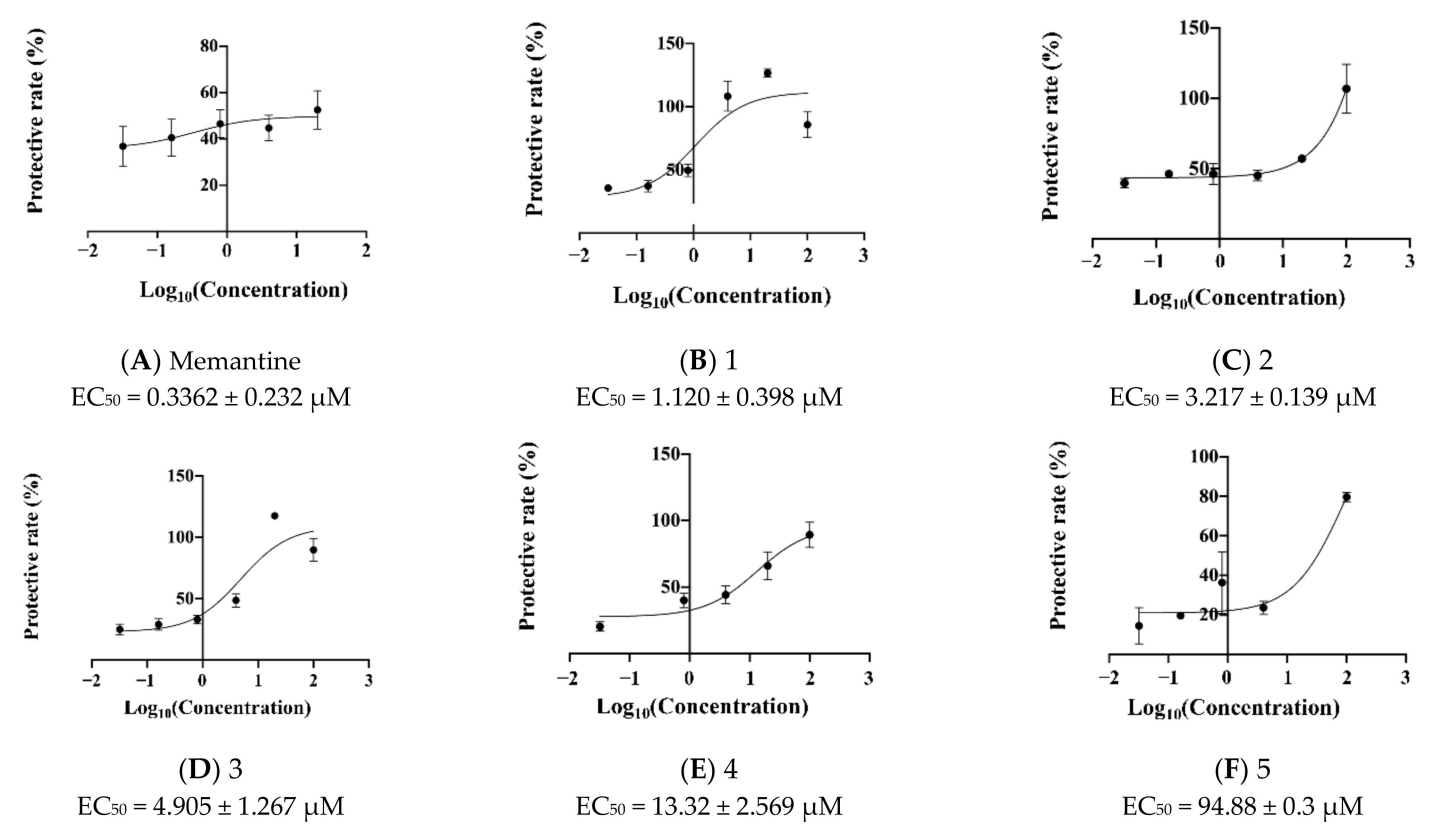

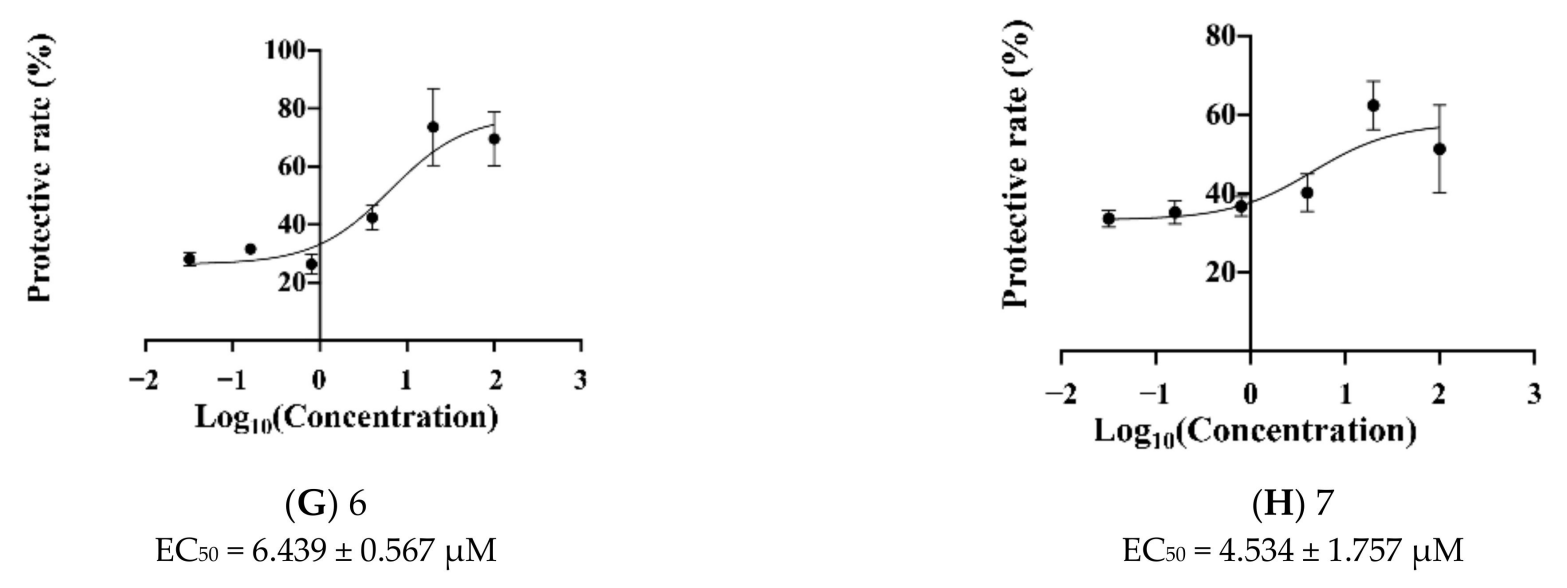
Value of EC_50_ of novel structural derivatives of memantine against copper-induced toxicity. (**A**–**H**) Dose-dependent curve of memantine (as a positive reference drug) (**A**) and memantine derivatives, including 1 (**B**) modafinil-memantine, 2 (**C**) piracetam-memantine, 3 (**D**) picamilon-memantine, 4 (**E**) glycyl-glycine-memantine, 5 (**F**) glycyl-glycyl-glycine-memantine 6 (**G**) sarcosine-memantine, 7 (**H**) dimethylglycine-memantine. Data are expressed as mean ± SEM, *n* = 3.

As shown in Figure 3, 300 μΜ copper significantly reduced the cell viability of APPswe cells as compared to the control group (Figure 3A–H, all *p* < 0.001 vs. control group). Seven new compounds improved the cell viability of copper-injured APPswe cells with various effects (Figure 3A–H, *p* < 0.05–0.001). Among compounds **1** to **7**, modafinil-memantine, piracetam-memantine, picamilon-memantine, glycyl- glycine-memantine, glycyl-glycyl-glycine-memantine and dimethylglycine-memantine displayed neuroprotective effects comparable with the positive drug memantine in the AD cell model (Figure 3A–H, *p* < 0.05–0.001). Meanwhile, compounds **1**–**7** showed a good dose-dependent with EC_50_ values ranging from 1.120 μΜ to 94.88 μΜ (Figure 4A–H). Compounds modafinil-memantine (EC_50_ = 1.120 ± 0.398 μΜ), piracetam-memantine (EC_50_ = 3.217 ± 0.139 μΜ), picamilon-memantine (EC_50_ = 4.905 ± 1.267 μΜ), sarcosine-memantine (EC_50_ = 6.439 ± 0.567 μΜ), and dimethylglycine-memantine (EC_50_ = 4.534 ± 1.757 μΜ) were potential anti-AD candidates with good EC_50_ value, especially compound modafinil-memantine. However, compounds modafinil-memantine and picamilon-memantine at 100 μΜ exhibited cell cytotoxicity, which was similar to the positive drug memantine at 100 μΜ (Figure 3B,D).

Taken together, these novel structural derivatives of memantine improved cell viability against copper-induced toxicity in APPswe cells, indicating that these active compounds may exert neuroprotective effects from Aβ toxicity in AD.

### 2.3. Novel Structural Derivatives of Memantine Improve Cell Viability against Glutamate-Induced Neurotoxicity in SH-SY5Y Cells

**Figure 5:**
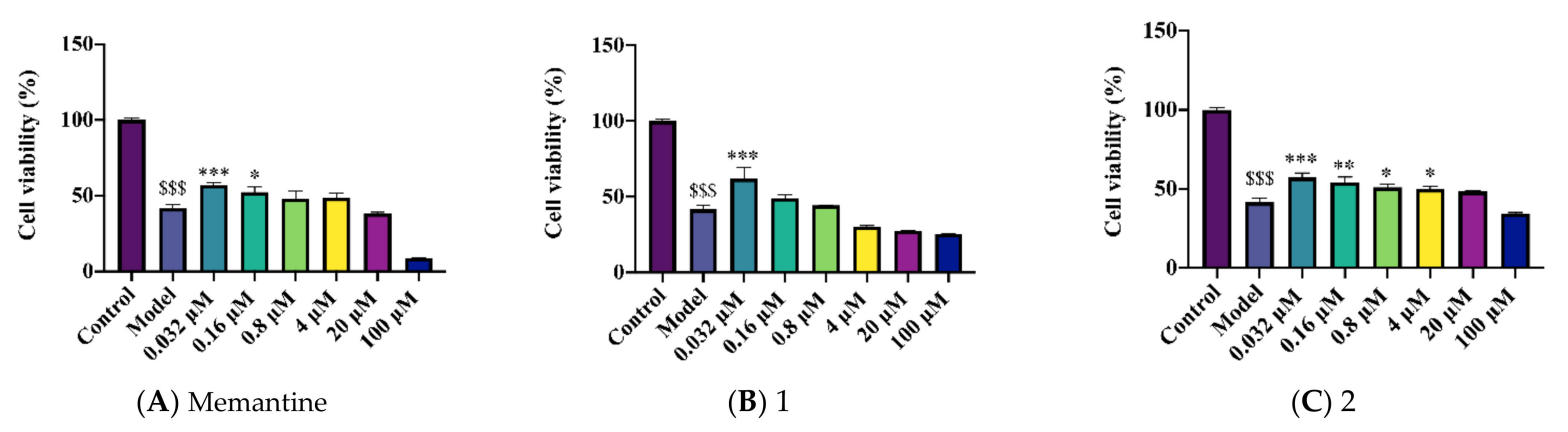

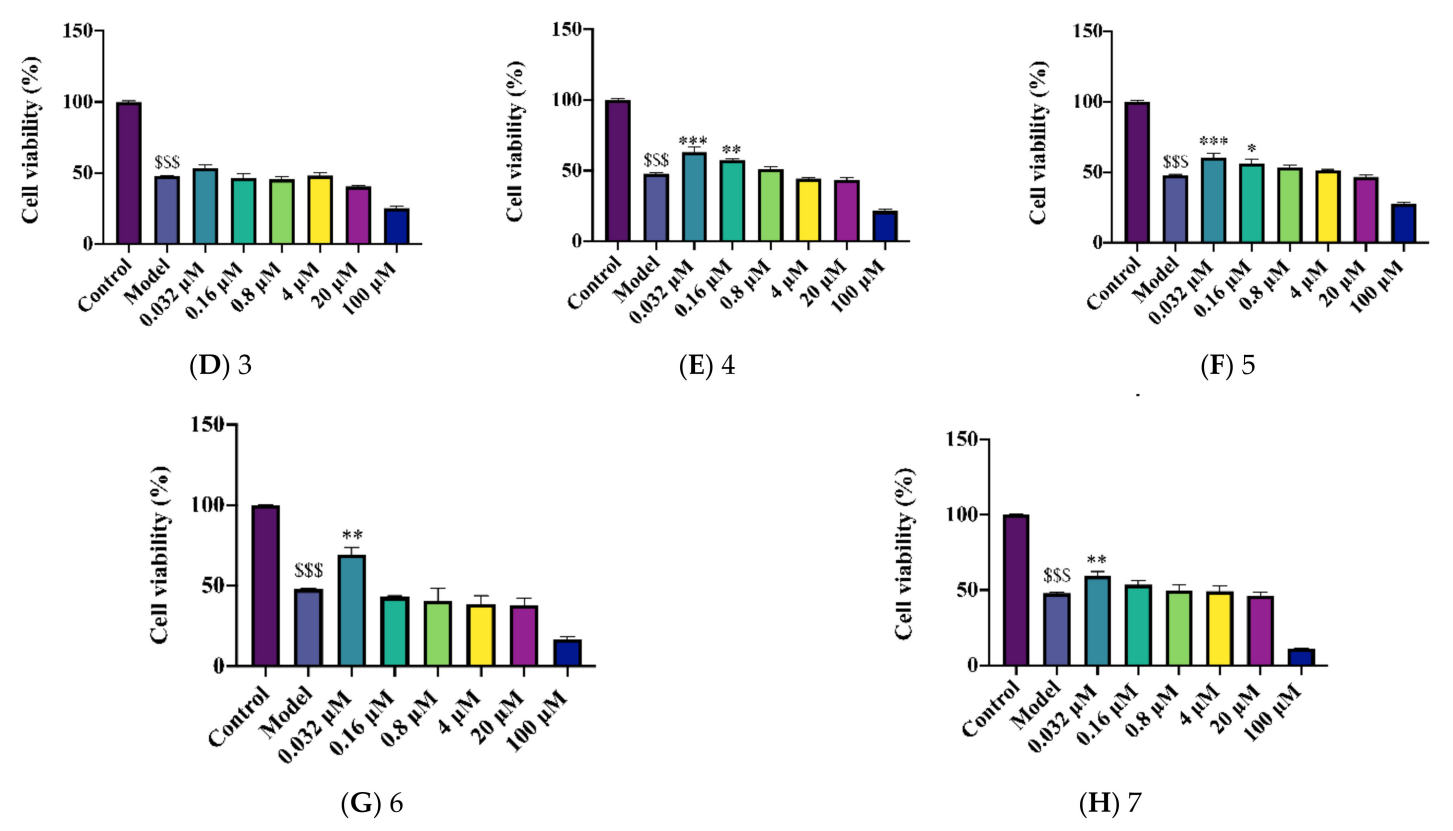
Cell viability of novel structural derivatives of memantine in glutamate-injured SH-SY5Y cells. (**A**–**H**) Cell viability of memantine (as a positive drug) (**A**) and memantine derivatives, including 1 (**B**) modafinil-memantine, 2 (**C**) piracetam-memantine, 3 (**D**) picamilon-memantine, 4 (**E**) glycyl-glycine-memantine, 5 (**F**) glycyl-glycyl-glycine-memantine, 6 (**G**) sarcosine-memantine, 7 (**H**) dimethylglycine-memantine, using MTS assay. Data are expressed as mean ± SEM, *n* = 3. ^$$$^ *p* < 0.001 vs. the control group; * *p* < 0.05, ** *p* < 0.01, *** *p* < 0.001 vs. the model.

Glutamate is an excitatory neurotransmitter and participates in the plasticity of the central nervous system diseases affecting cognition, memory, and learning. Neuronal cell loss associated with glutamate neurotoxicity is a close pathological event in AD. The results showed that glutamate significantly decreased the cell viability of SH-SY5Y cells (Figure 5A–H, all *p* < 0.001). These compounds treated ranging from 0.032 μΜ to 4 μΜ showed different degrees of neuroprotection effects. Compound piracetam-memantine treated at 0.032 μΜ, 0.16 μΜ, 0.8 μΜ, and 4 μΜ significantly improved the cell viability of SH-SY5Y cells, and compound glycyl-glycine-memantine treated at 0.032 μΜ and 0.16 μΜ increased cell viability (Figure 5E, *p* < 0.01–0.001). These two compounds have a similar neuroprotective effect to the positive drug memantine. Compounds glycyl-glycyl-glycine-memantine, sarcosine-memantine, dimethylglycine-memantine, treated at 0.032 μΜ increased cell viability (Figure 5F–H, *p* < 0.05–0.01). Combined, these active compounds may have neuroprotective effects with a correlation with the glutamate receptors.

### 2.4. Prediction of EC_50_ Using HYBOT Descriptors

**Figure 6:**
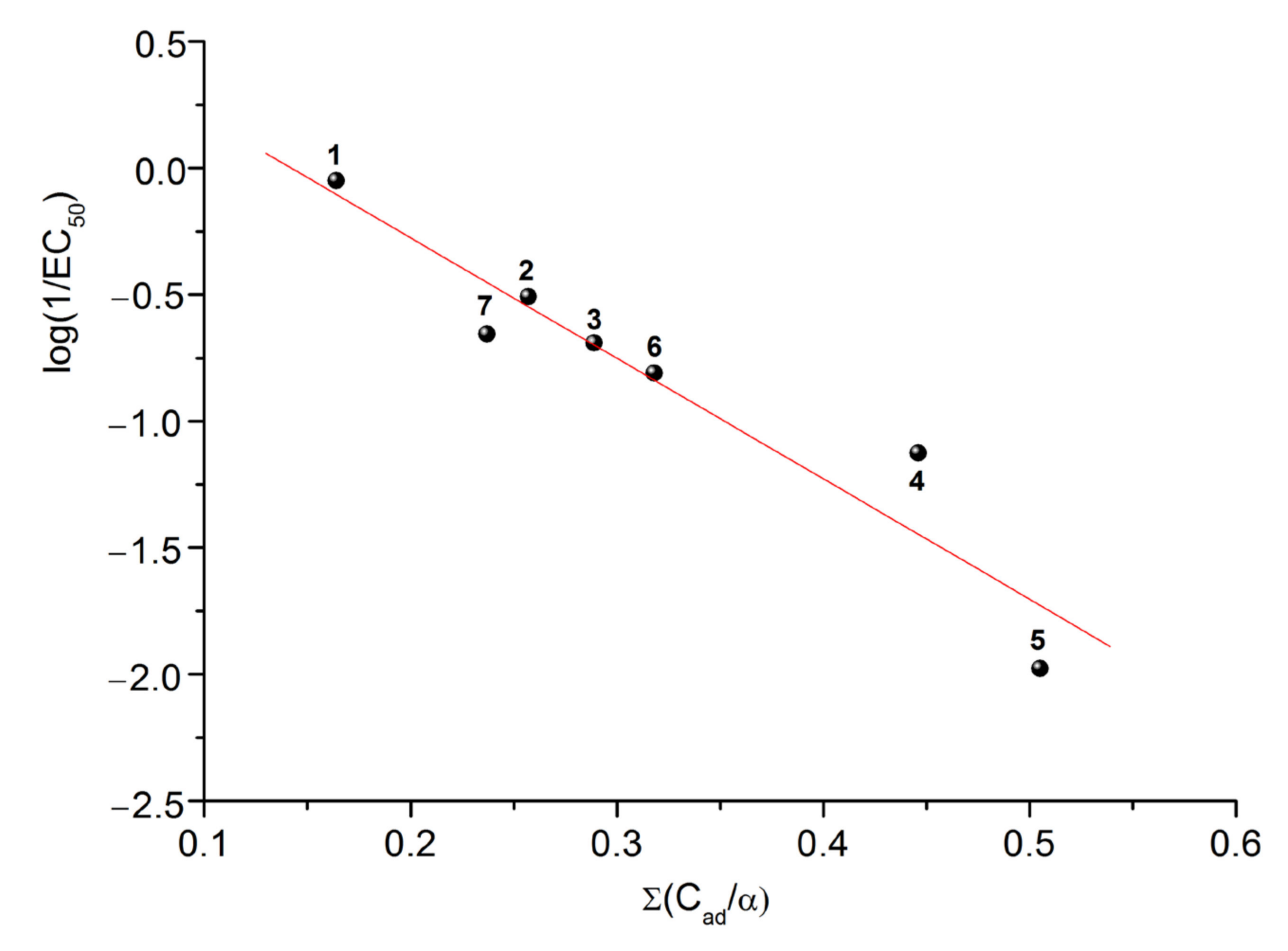
Dependence of $\log(1/EC_{50})$ versus $\Sigma (C_{ad}/\alpha )$ descriptor (compounds numbering corresponds to Figure 2).

One of the main tasks of the QSAR models is to predict the biological properties of the systems. In this work, we attempted to develop a correlation model based on the HYBOT (H-bond thermodynamics) descriptors in order to predict EC_50_. To this end, all the physicochemical descriptors available in the software package were used. As a result of the fitting, the best correlation was obtained with the descriptor characterizing the sum of the donor and acceptor ability of atoms in a molecule to form hydrogen bonds normalized to molecular polarizability ($\Sigma (C_{ad}/\alpha )$) (Figure 6). The final correlation dependence can be described by the following equation:

(1) $$\log(1/EC_{50})=(0.677\pm 0.236)-(4.762\pm 0.703)\cdot \Sigma (C_{ad}/\alpha )$$

*R* = 0.9496; *SD* = 0.21; *n* = 7; *F* = 45.9

Evidently, the $\Sigma (C_{ad}/\alpha )$ descriptor allows for all types of the interactions of the studied molecules with the biological environment: specific (*C_ad_*) and nonspecific (*α*). Thus, it is possible to predict the EC_50_ values for a given class of compounds with the help of Equation (1) based only on the structural formula without the expensive biological experiments.

### 2.5. Solubility Experiments

**Figure 7:**
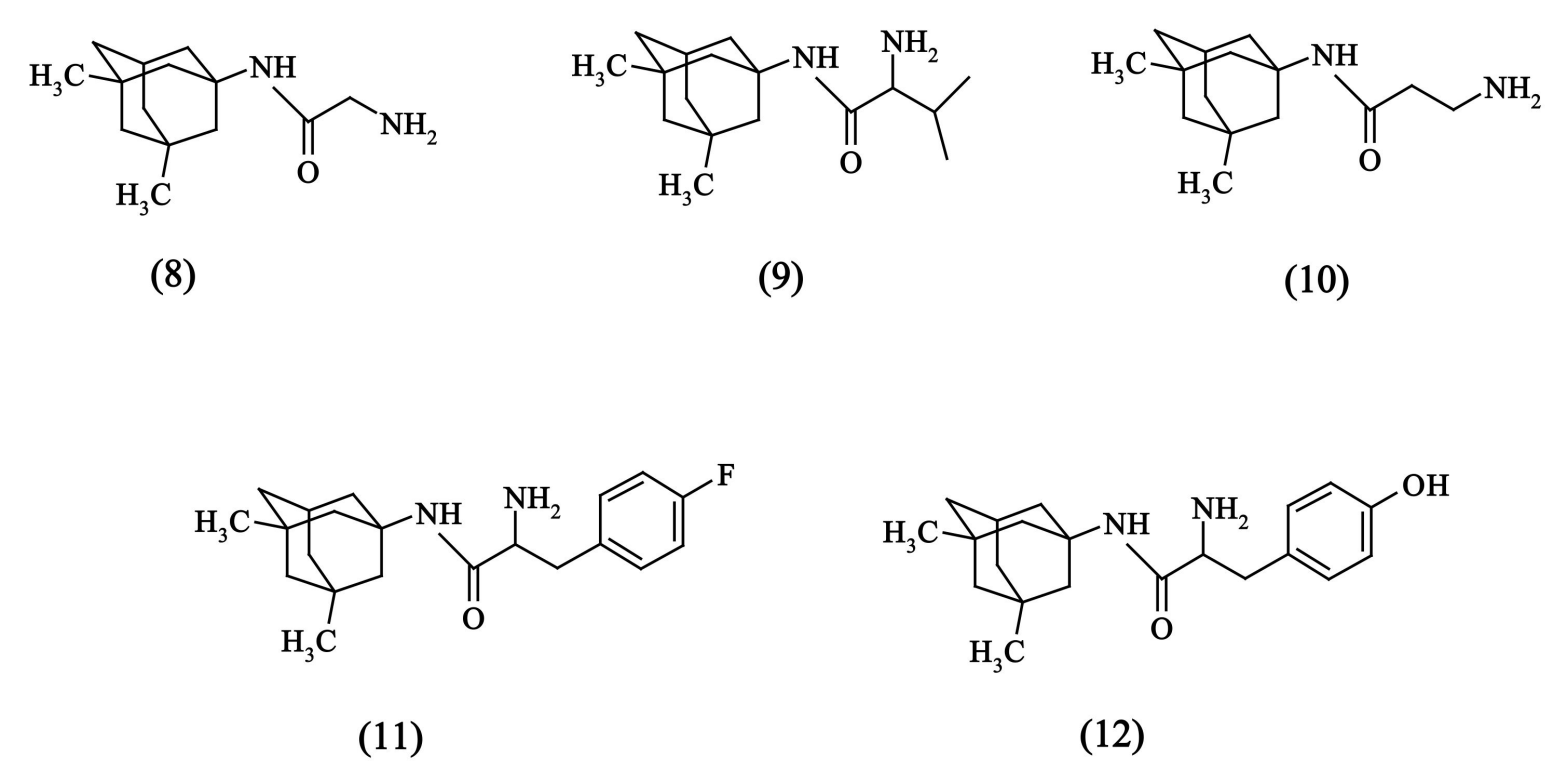
Formulas of the compounds studied in the previous study [30] structurally analogous to the derivatives investigated in the present work (see Figure 2 for the structures): **8**—glycine-memantine; **9**—valine-memantine; **10**—beta-alanine-memantine; **11**—4-F-phenylalanine-memantine; **12**—tyrosine-memantine.

Both the objects of this investigation (1–7), represented in Figure 2, and the structural analogues from the previous study [32] (8–12), illustrated in Figure 7, were taken into consideration.

**Figure 8:**
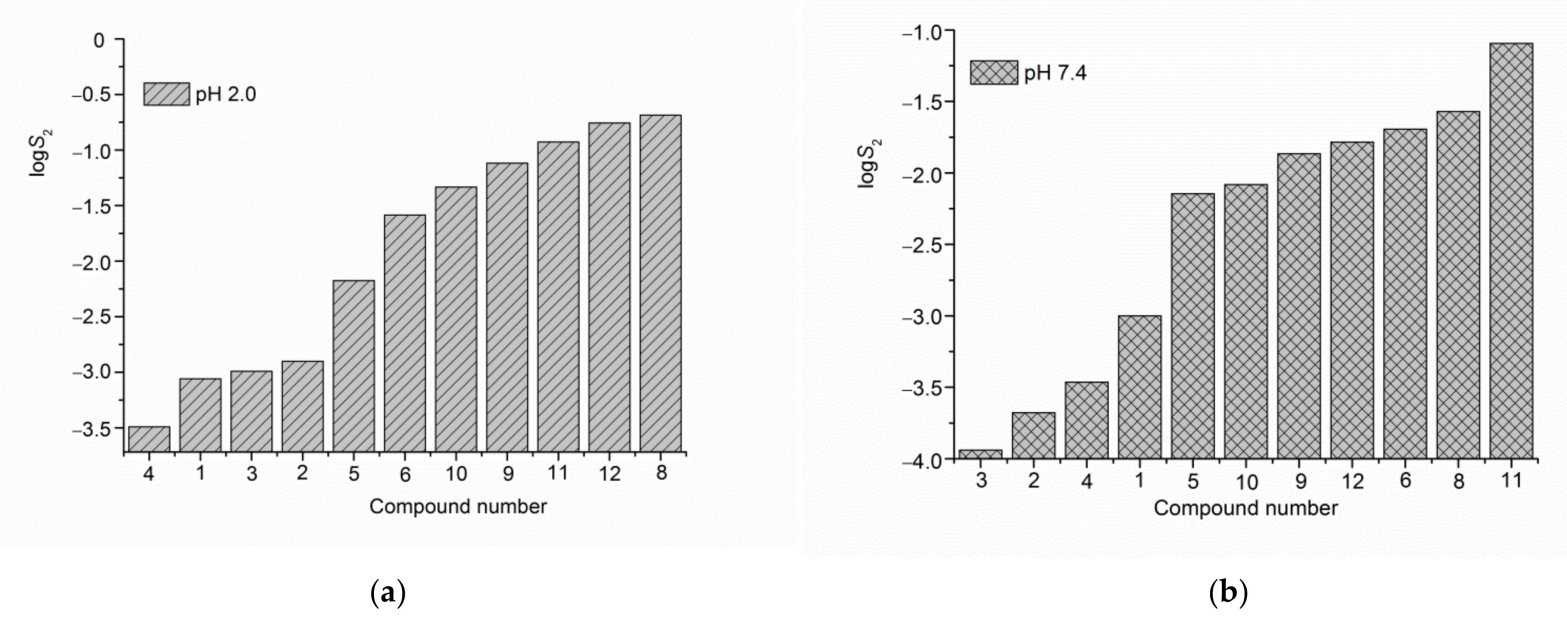
Solubility of the considered compounds in buffer solutions pH 2.0 (**a**) and pH 7.4 (**b**) at 298.15 K (compounds numbering corresponds to Figure 2 and Figure 7).

The solubility of compounds (**1**)–(**6**) was determined in buffer solutions at pH 2.0 and pH 7.4 simulating the gastric juice and the blood plasma/jejunum/ileum media at 298.15 K. The solid residuals after the dissolution experiments were isolated and analyzed. The comparison did not show any changes of the crystalline phases before and after the solubility tests (Figure 8). The solubility results for the studied derivatives are represented in Table 1 together with those of the compounds reported previously. For better visualization, the data are illustrated as a diagram in an ascending order of the solubility values (Figure 8).

As follows from the experiments, for the majority of the substances—(2), (3), (8), (9), (10), (11), (12)—the solubility in buffer solution pH 2.0 is essentially higher than in the medium of pH 7.4. The difference is the greatest for tyrosine-substituted compound (**12**). For piracetam—(2), valine—(9), and β-alanine—(10) derivatives these differences are very close (5.95 ÷ 5.57-fold). In their turn, compounds (**1**), (**4**), (**5**) and (**6**) have practically the same solubility values in both studied pHs. Appealing to the structures of the substances (Figure 2 and Figure 7), it can be proposed that a higher solubility in muriatic buffer pH 2.0 is a result of the stronger ionization (protonation) of the molecules in the acidic solution. On the other hand, the compounds revealing the close solubilities in both investigated media are intended to be in the same ionization state. Some difference in the solubility (between pH 2.0 and pH 7.4) of compounds (**1**), (**4**), (**5**) and (**6**) can also be attributed to the impact of the buffer components in the aqueous solutions. The same order of the solubility of these substances in both pHs (S_2_(4) < S_2_(1) < S_2_(5) < S_2_(6)) proves the above proposal. It should be emphasized that if we consider all the compounds, the sequence of the solubility values depends on the pH. In buffer pH 2.0: S_2_(4) < S_2_(1) < S_2_(3) < S_2_(2) < S_2_(5) <S_2_(6) < S_2_(10) <S_2_(9) < S_2_(11) < S_2_(12) < S_2_(8), whereas at pH 7.4 the order is: S_2_(3) < S_2_(2) < S_2_(4) < S_2_(1) < S_2_(5) < S_2_(10) < S_2_(9) < S_2_(12) < S_2_(6) < S_2_(8) < S_2_(11). Obviously, the mutual influence of the structural fragments in the molecules impact greatly the solubility behavior. So, at the next step it was interesting to trace this influence. The analysis of the substituent impact on the solubility of the memantine derivatives studied in the present work at 298.15 K led to the following conclusions.

The addition of the hydrophilic NH-CO- fragment to compound (**4**) resulting in compound (**5**), expectedly, enhances the solubility in 20.8-fold in both pHs. The replacement of the NH_2_- group in compound (**10**) by the fragment containing the CH_2_-NH-CO-pyridine ring to obtain compound (**3**) results in 45.3-fold and 72.2-fold solubility decrease (in pH 2.0 and pH 7.4, respectively), probably, due to a higher hydrophilicity of the NH_2_- group as compared to the NH- one, and addition of a bulky pyridine ring. Expectedly, the solubility reduction is less in pH 2.0 as compared to pH 7.4 due to the possibility of the nitrogen atom protonation. Taking into account the presence of the two bulky hydrophobic phenyl substituents in compound (**1**), very low solubility in aqueous medium can be expected. But, probably, the sulfinyl (SO) functional group in (1) molecule (capable of the hydrogen bonding) makes the solubility higher than for compound (**4**) in pH 2.0 and compounds (**2**), (**3**) and (**4**) in pH 7.4.

**Figure 9:**
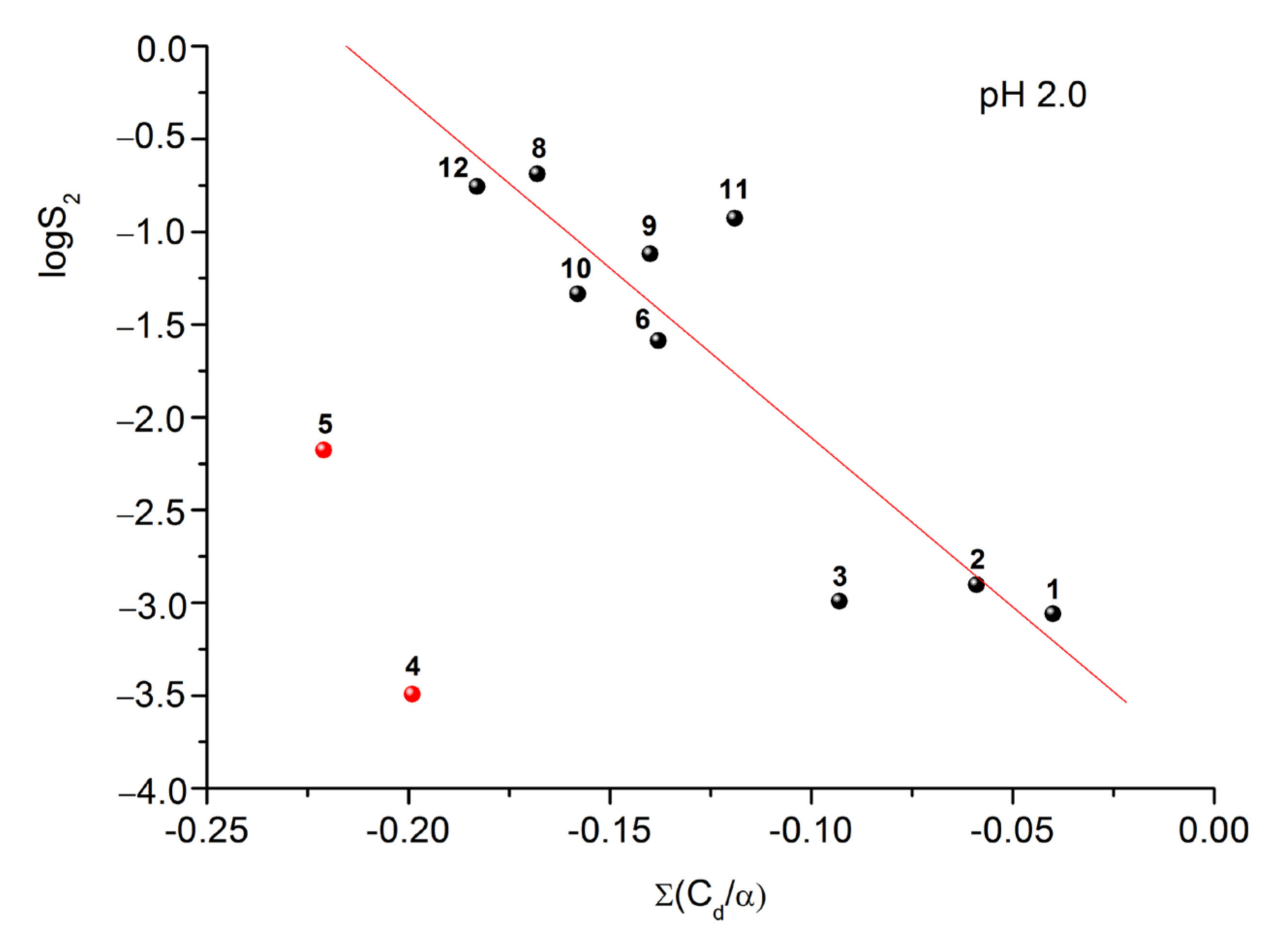
Dependence of $\log(S_{2}^{})$ versus $\Sigma (C_{d}/\alpha )$ descriptor. (compound numbering corresponds to Figure 2 and Figure 7).

Since the solubility is an important physicochemical characteristic of a substance which defines the bioavailability of drugs, predicting the solubility values for this class of compounds was one of the goals of the study. To carry out the correlation analysis, we used the physicochemical descriptors HYBOT as the independent variables [34]. In turn, the logarithms of the solubility values were chosen as the dependent ones. Analysis of 32 independent descriptors from the HYBOT program showed that the solubility values in buffer pH 2.0 correlate well with the parameter describing the sum of the donor ability of atoms in a molecule to form hydrogen bonds normalized to polarizability ($\Sigma (C_{d}/\alpha )$). The results of the analysis are presented in Figure 9. If we neglect the experimental points (4) and (5) (which deviate significantly from the general dependence), then the correlation equation can be represented as follows:

(2) $$\log(S_{2}^{pH2.0})=(3.94\pm 0.44)-(18.3\pm 3.4)\cdot \Sigma (C_{d}/\alpha )$$

*R* = 0.8991; *SD* = 0.47; *n* = 9; *F* = 29.5

**Figure 10:**
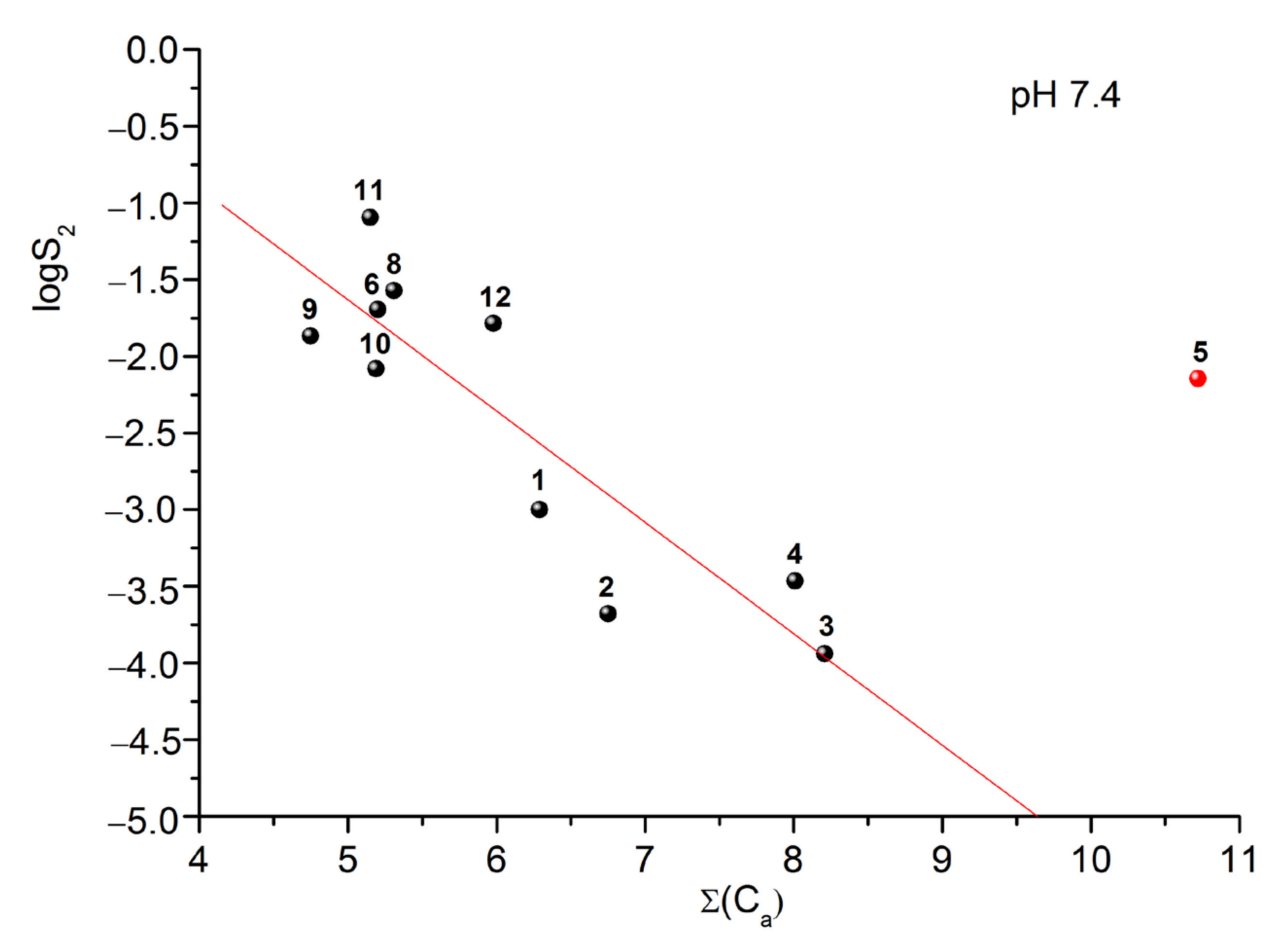
Dependence of $\log(S_{2}^{pH7.4})$ versus $\Sigma (C_{d}/\alpha )$ descriptor. (compound numbering corresponds to Figure 2 and Figure 7).

**Figure 11:**
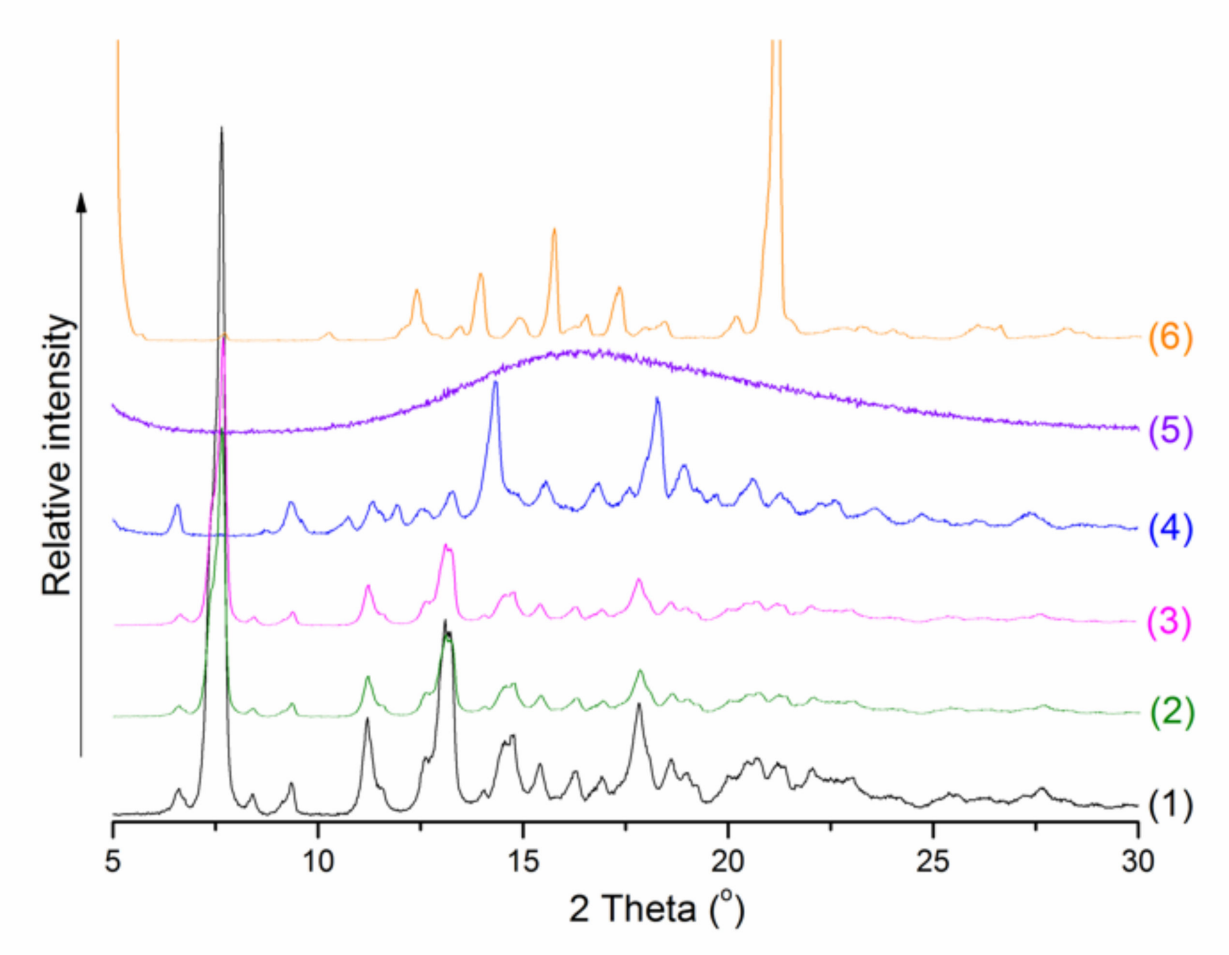
PXRD patterns of compounds (**1**–**6**). (compounds numbering corresponds to Figure 2 and Figure 7).

In turn, if we analyze the solubility in buffer solution pH 7.4, we can disclose a relationship between these values and the descriptor characterizing the sum of the acceptor ability of the atoms in a molecule to form hydrogen bonds ($\Sigma (C_{a})$). The results are given in Figure 10. As in the previous case, the experimental point for compound (**5**) deviates significantly from the observed dependence, which may be due to the amorphous state of the substance in the bottom phase (Figure 11), which leads to an overestimated solubility as compared to if it would be in the crystalline state. If we neglect of this value, then the correlation equation can be represented as follows:

(3) $$\log(S_{2}^{pH7.4})=(2.00\pm 0.84)-(0.726\pm 0.136)\cdot \Sigma (C_{a})$$

*R* = 0.8839; *SD* = 0.50; *n* = 10; *F* = 28.6

Analysis of Equations (2) and (3) shows that in buffer solution at pH 2.0, the solubility values are sensitive both to the donor ability of the substance to form hydrogen bonds (specific interactions) and to the polarizability of the molecule (nonspecific interactions). In contrast, in buffer pH 7.4 the dependence on the polarizability disappears, but the sensitivity to acceptor ability becomes evident. Apparently, this is due to different ionization states of the studied molecules in the solutions. The derived correlation dependences make it possible to predict the solubility of the studied class of compounds based only on their structural formulas. This fact greatly simplifies the task of designing the compounds with the improved solubility and enhanced bioavailability.

## 3. Materials and Methods

### 3.1. Materials

#### 3.1.1. Chemicals

Unless otherwise stated, the starting materials, reagents, and solvents were obtained from commercial purchase and used as supplied without further purification. Analytical thin-layer chromatography (TLC) was run on Merck silica gel 60 F-254, with detection by UV light (l j 254 nm). Memantine, amino acids, peptides, TBTU coupling regent, triethylamine and all necessary solvents for the synthesis were purchased from Sigma Aldrich. 2-Benzhydrylsulphinylacetic acid, (2-Oxopyrrolidin-1-yl)acetic Acid and 4-(Nicotinamido)butanoic acid were purchased from Shanghai Ruifu Chemical (Shanghai, China).

Identification of compounds

1H and 13C spectra were recorded on Bruker Avance II+ spectrometer (14.09 T magnet), operating at 600.11 MHz 1H frequencies, equipped with 5 mm BBO probe with z-gradient coil. The temperature is maintained at 293 K, using Bruker B-VT 3000 temperature unit with airflow of 535 L/h. All chemical shifts are reported in parts per million (ppm), referenced against teramethylsylane (TMS, 0.00 ppm) or using the residual solvent signal (7.27 ppm for CDCL3 of 2.5 ppm for DMSO). Electrospray mass spectrometry (ESI-MS) experiments were acquired on Bruker Compact QTOF-MS (Bruker Daltonics, Bremen, Germany) and controlled by the Compass 1.9 Control software. The data analysis was performed and the mono-isotopic mass values were calculated using Data analysis software v 4.4 (Bruker Daltonics, Bremen, Germany). The analyses were conducted in the positive ion mode at a scan range from *m*/*z* 50 to 1000, and nitrogen was used as nebulizer gas at a pressure of 4 psi and flow of 3 L/min^−1^ for the dry gas. The capillary voltage and temperature were set at 4500 V and 493 K, respectively. An external calibration for mass accuracy was carried out by using of sodium formate calibration solution. The precursor ion of each compound was selected, and ESI–MS/MS analysis was performed by collision-induced dissociation (CID); nitrogen was the collision gas, and the collision energy varied from 5 to 40 eV. MSn experiments were conducted on an ion trap instrument Esquire 3000 (Bruker Daltonics, Bremen, Germany) and controlled by the Esquire Control 5.3.11 software. ESI-MS data were collected in positive-ion mode at a scan range from *m*/*z* 50 to 500. In all ESI-MS measurements, the nebulizer gas pressure was 124.1 kPa at a flow rate of 5 L min^−1^; the desolvation temperature was 573 K and capillary voltage was adjusted to 4000 V. The sample solutions were delivered to nebulizer by a syringe pump (Cole Parmer, Vernon Hills, IL, USA) at a flow rate 3 µL min^−1^.

#### 3.1.2. Solvents

Phosphate buffer pH 7.4 (I = 0.15 mol·L^−1^) contained KHPO_4_ (9.1 g in 1 L) and NaH_2_PO_4_·12H_2_O (23.6 g in 1 L). Buffer solution pH 2.0 (I = 0.10 mol·L^−1^) was prepared as follows: 6.57 g KCl was dissolved in water, and 119.0 mL of 0.1 mol·L^−1^ hydrochloric acid added. The volume was adjusted to 1 L with water. The pH value was controlled with a pH meter (Five GoTM F2, Mettler Toledo) using pH 4 and pH 7 standards. Bidistilled water (electrical conductivity equal to 2.1 μS cm^−1^) was used throughout the experiments. The reagents were used as received without purification.

#### 3.1.3. Cell Culture and Treatment

SH-SY5Y cells (ATCC, Manassas, VA, USA) were cultured in the Dulbecco’s Modified Eagle Medium (DMEM) supplemented with 10% fetal bovine serum (FBS; Gibco/Invitrogen, Grand Island, NY, USA). SH-SY5Y cells transfected with the Swedish mutant form of human APP (referred to as “APPswe cells”) is an established cell model of AD [35] in which copper triggers Aβ overproduction, cultured in Dulbecco’s Modified Eagle Medium/Nutrient Mixture F-12 (DMEM/F12) supplemented with 2 mM L-glutamine, 10% fetal bovine serum (FBS; Gibco/Invitrogen, Grand Island, NY, USA), and 400 μM G418 (Sigma Chemical Company, St. Louis, MO, USA).

### 3.2. Chemical Synthesis

Amide bond formation for all seven compounds was realized according to method described by Knorr et al. [31]. Memantine (8 mmol) was dissolved in 5 mL DCM, TEA (8 mmol) was added to the solution. In separate flask the carboxyl component (8.8 mmol) was dissolved in 10 mL DCM. To the mixture was added TEA (8.8 mmol) and TBTU (12.3 mmol). Both solutions were mixed together after 30 min. After 24 h the reaction mixture was washed by 5% NaHCO_3_ solution twice, then dried with anhydrous Na_2_SO_4_. Sarcosine and the peptides necessitated Boc-protection group removing (DCM/TFA 50:50 for 3 h) before any further purification. The obtained compound was purified by flash chromatography—system TCM/MeOH 97:3 or Hexan/EtOAc 5:4 ratio, depending of the polarity of the compounds.

Modafinil-memantine (1); ^1^H NMR (500.17 MHz, DMSO-d6), δ, ppm: 0.58 (s, 6H, 2CH3), 0.88(s, 2H, AdmH), 1.00–1.15 (m, 4H, AdmH), 1.40 (m, 4H, AdmH), 1.59 (m, 2H, AdmH), 1.84 (q, 1H, AdmH), 3.02–3.27 (m, 2H, CH2, ModH), 5.15 (s, CH, ModH), 7.11–7.18 (m, 2H, ModH), 7.18–7.24 (m, 4H, ModH), 7.34–7.39 (m, 4H, ModH), 7.54 (s, NH, ModH).

^13^C NMR (125.77 MHz, DMSO-d6), δ, ppm: 29.14 (2CH3), 29.45 (CH, AdmC), 31.78 (C, AdmC),34.39(C, AdmC), 39.42 (CH2, AdmC), 42.31 (2CH2, AdmC), 46.93 (2CH2, AdmC), 50.22 (C, AdmC), 53.17 (C, AdmC), 56.79 (CH2, ModH), 69.25 (CH, ModH), 128.32 (6C, para + orto, ModH), 129.89 (4C, meta, ModH), 134.19–138-42 (2C, ModH), 161.86 (C=O, ModH). Yield: 65%.

ESI-MS; *m*/*z* 436.63 [M + H]+.

Piracetam-memantine (2); ^1^H NMR (500.17 MHz, DMSO-d6), δ, ppm: 0.58 (s, 6H, 2CH3), 0.88(s, 2H, AdmH), 1.00–1.15 (m, 4H, AdmH), 1.41 (m, 4H, AdmH), 1.59 (m, 2H, AdmH), 1.75 (q, 2H, ring, PiracH), 1.84 (q, 1H, AdmH), 2.02 (t, 2H, ring, PiracH), 3.21 (m, 2H, ring, PiracH), 3.62 (s, 2H, PiracH), 7.17 (s, NH, PiracH).

^13^C NMR (125.77 MHz, DMSO-d6), δ, ppm: 17.49 (CH2, ring, PiracC), 29.34 (2CH3, AdmC), 29.90 (CH, AdmC), 29.90 (CH2, ring, PiracC), 31.81 (C, AdmC), 34.39(C, AdmC), 39.60 (CH2, AdmC), 42.31 (2CH2, AdmC), 45.26 (CH2, PiracC), 47.09 (CH2, ring, PiracC), 47.09 (2CH2, AdmC), 50.28 (CH2, AdmC), 52.64 (C, AdmC), 161.86 (C=O, PiracC), 174.26 (C=O, ring, PiracC). Yield: 60%.

ESI-MS; *m*/*z* 305.44 [M + H]+.

Picamilon-memantine (3); ^1^H NMR (500.17 MHz, DMSO-d6), δ, ppm: 0.57 (s, 6H, 2CH3), 0.87(s, 2H, AdmH), 1.00–1.15 (m, 4H, AdmH), 1.41 (m, 4H, AdmH), 1.59 (m, 2H, AdmH), 1.63 (m, 2H, CH2, PicaH), 1.83 (q, 1H, AdmH), 1.98 (t, 2H, CH2, PicaH), 3.17 (qt, 2H, CH2, PicaH), 7.11 (s, NH, MemH), 7.29 (qt, 1H, ring, PicaH), 7.79 (s, NH, MemH), 8.05 (qt, 1H, ring, PicaH), 8.50 (m, 1H, ring, PicaH), 8.89 (m, 1H, ring, PicaH).

^13^C NMR (125.77 MHz, DMSO-d6), δ, ppm: 25.48 (CH2, PicaC), 29.27 (2CH3, AdC), 29.60 (CH, AdmC), 31.35 (C, AdmC), 34.35 (CH2, PicaC), 34.39(C, AdmC), 39.41 (CH2, AdmC), 39.41 (CH2, PicaC), 42.42 (2CH2, AdmC), 47.12 (2CH2, AdmC), 50.32 (CH2, AdmC), 52.30 (C, AdmC), 123.20 (CH, ring, PicaC), 130.26 (C, ring, PicaC), 134.59 (CH, ring, PicaC), 148.45 (CH, ring, PicaC), 151.67 (CH, ring, PicaC), 161.85 (C=O, PicaC), 171.44 (C=O, PicaC). Yield: 58%.

ESI-MS; *m*/*z* 370.51 [M + H]+.

Glycyl-glycine-memantine (4); ^1^H NMR (500.17 MHz, DMSO-d6), δ, ppm: 0.81 (s, 6H, 2CH3), 1.10 (s, 2H, AdmH), 1.20–1.34 (m, 4H, AdmH), 1.56 (q, 4H, AdmH), 1.75 (m, 2H, AdmH), 2.07 (m, 1H, AdmH), 3.37 (2H, NH2), 3.71 (2H, CH2), 3.84 (2H, CH2), 7.48 (2H, NH2), 8.46 (1H, NH).

^13^C NMR (125.77 MHz, DMSO-d6), δ, ppm: 29.96 (CH, AdmC), 30.58 (2CH3, AdmC), 32.30 (C, AdmC), 32.31 (C, AdmC), 40.06 (CH2, AdmC), 42.77 (CH2), 42.95 (CH2), 43.01 (2CH2, AdmC), 47.54 (2CH2, AdmC), 50.71 (CH2, AdmC), 52.91 (C, AdmC), 166.87 (C=O), 167.59 (C=O). Yield: 76%.

ESI-MS; *m*/*z* 294.41 [M + H]+.

Glycyl-glycyl-glycine-Memantine (5); ^1^H NMR (500.17 MHz, DMSO-d6), δ, ppm: 0.81 (s, 6H, 2CH3), 1.10 (s, 2H, AdmH), 1.20–1.34 (m, 4H, AdmH), 1.36–1.48 (CH2), 1.56 (q, 4H, AdmH), 1.75 (m, 2H, AdmH), 2.07 (m, 1H, AdmH), 3.36 (1H, NH) 3.62 (2H, CH2), 3.82 (2H, CH2), 7.32 (1H, NH), 7.42 (2H, NH2), 8.66 (1H, NH).

^13^C NMR (125.77 MHz, DMSO-d6), δ, ppm: 30.12 (CH, AdmC), 30.54 (2CH3, AdmC), 32.39 (C, AdmC), 32.41 (C, AdmC), 39.90 (CH2, AdmC), 42.00 (CH2), 42.70 (CH2), 43.00 (2CH2, AdmC), 46.50 (CH2), 47.60 (2CH2, AdmC), 50.68 (CH2, AdmC), 52.86 (C, AdmC), 166.84 (C=O), 167.97 (C=O), 168.86 (C=O). Yield: 61%.

ESI-MS; *m*/*z* 351.46 [M + H]+.

Methylglycine-memantine (6); ^1^H NMR (500.17 MHz, DMSO-d6), δ, ppm: 0.83 (s, 6H, 2CH3), 1.12 (s, 2H, AdmH), 1.22–1.36 (m, 4H, AdmH), 1.58 (q, 4H, AdmH), 1.78 (m, 2H, AdmH), 2.09 (m, 1H, AdmH), 2.54 (3H, CH3), 3.35 (1H, NH), 3.59 (2H, CH2), 8.67 (1H, NH)

^13^C NMR (125.77 MHz, DMSO-d6), δ, ppm: 29.94 (CH, AdmC), 30.48 (2CH3, AdmC), 32.31 (C, AdmC), 32.32 (C, AdmC), 33.06 (CH3), 39.58 (CH2, AdmC), 42.63 (2CH2, AdmC), 47.38 (2CH2, AdmC), 49.84 (CH2) 50.53 (CH2, AdmC), 53.43 (C, AdmC), 164.47 (C=O). Yield: 86%.

ESI-MS; *m*/*z* 251.39 [M + H]+.

Dimethylglycine-memantine (7); ^1^H NMR (500.17 MHz, DMSO-d6), δ, ppm: 0.82 (s, 6H, 2CH3), (s, 2H, AdmH), 1.21–1.35 (m, 4H, AdmH), 1.63 (q, 4H, AdmH), 1.95 (m, 2H, AdmH)), 2.03 (m, 1H, AdmH), 2.78 (3H, CH3), 3.82 (2H, CH3), 8.27.

^13^C NMR (125.77 MHz, DMSO-d6), δ, ppm: 29.20 (CH, AdmC), 30.49 (2CH3, AdmC), 32.33 (C, AdmC), 32.31 (C, AdmC), 36.24 (CH3), 41.26 (CH2, AdmC), 43.37 (2CH2, AdmC), 47.39 (2CH2, AdmC), 49.83 (CH2) 52.12 (CH2, AdmC), 58.25 (C, AdmC), 163.56 (C=O). Yield: 82%.

ESI-MS; *m*/*z* 265.41 [M + H]+.

### 3.3. Measurement of Neuroprotective Effects against Copper-Induced Toxicity in APPswe Cells

APPswe cells were seeded into 96-well plates at a density of 5 × 104 cells/well. APPswe cells were divided into three groups: control group (DMEM/F12 medium), copper-injured group (300 μM copper; model group), and copper-injured groups treated with compounds, in which cells were treated with 0.032 μΜ, 0.16 μΜ, 0.8 μΜ, 4 μΜ, 20 μΜ, and 100 μΜ tested compounds. The cell viability of APPswe cells was measured at 36 h incubation by MTS assay. The test was made and repeated four times.

### 3.4. Measurement of Neuroprotective Effects against Glutamate-Induced Toxicity in SH-SY5Y Cells

SH-SY5Y cells were seeded into 96-well plates at a density of 5×10^4^ cells/well. Cells were divided into three groups: control group (DMEM medium), glutamate-injured group (7 mM glutamate; model group), and glutamate-injured groups treated with compounds, in which model groups were treated with 0.032 μΜ, 0.16 μΜ, 0.8 μΜ, 4 μΜ, 20 μΜ, and 100 μΜ tested compounds. The cell viability of SH-SY5Y cells was measured at 48 h incubation by MTS assay. The test was made and repeated four times.

### 3.5. Cell Viability Assay

Cell viability was assessed using an MTS assay and measured with a Spark 20M multimode microplate reader (Tecan Group Ltd., Mannedorf, Switzerland) at a wavelength of 490 nm. Cell viability = (OD compounds − OD blank)/(OD control − OD blank) × 100% (OD compounds: absorbance value of tested compounds group; OD control: absorbance value of control group; OD blank: absorbance value of culture medium without cells). Cell protection rate = (cell viability of compounds treatment groups—cell viability of model group)/cell viability of model group×100%.

### 3.6. Statistical Analysis

Data represent means ± standard error of the mean (SEM). Data were analyzed using a one-way ANOVA. *p*-values < 0.05 were considered statistically significant. Analysis was performed using GraphPad Prism Version 8.0 software (GraphPad Prism Software; La Jolla, CA, USA).

### 3.7. Solubility Determination

The standard shake-flask isothermal saturation method [36] was applied to the solubility experiments. The saturated solutions of the studied compounds were prepared in the glass screw-capped vials using the respective buffer solution (pH 2.0 or pH 7.4) which was vigorously stirred at a constant temperature of 298.15 K. The time required for reaching the equilibrium between the solute and the solvent was estimated with the help of the solubility kinetic dependences and was stated as no less than 24 h. After this time period, the saturated solutions stayed in the thermostat for no less than 5 h and then the solution was separated from the solid residual via centrifugation (Biofuge pico, Thermo Electron LED GmbH, Germany) at 298.15 K for 20 min at 10,000 rpm. The concentrations of the compounds in the saturated solutions were measured with the help of a spectrophotometer (Cary-50, Varian, USA) and using the calibration curves for each compound in each buffer at the appropriate wavelength. The experimental results were reported as an average of at least three replicated experiments.

### 3.8. Powder X-ray Diffraction (PXRD) Were Recorded According to SUROV et al. [37]

The powder XRD data of the bulk materials were recorded under ambient conditions on a D2 Phaser (Bragg-Brentano) diffractometer (Bruker AXS, Germany) with a copper X-ray source (λCuKα1 = 1.5406 Å) and a high-resolution position-sensitive LYNXEYE XE T detector. The samples were placed into the plate sample holders and rotated at a speed of 15 rpm during the data acquisition.

### 3.9. QSAR Modelling and Calculation of HYBOT Descriptors

QSAR models were constructed by partial least square regression (PLS) fitting (internal method of validation). Physicochemical HYBOT (Hydrogen Bond Thermodynamics) descriptors used as independent variables were calculated by the commercially available software HYBOT [32].

## 4. Conclusions

In this study, four memantine analogues of glycine derivatives, including glycyl-glycine, glycyl-glycyl-glycine, sarcosine, dimethylglycine and three conjugates with nootropics, modafinil, piracetam and picamilon which had favorable neuroprotective effects were synthesized for the first time.

The solubility of memantine analogues with nootropics and glycine derivatives in buffer solutions at pH 2.0 and pH 7.4 simulating the biological media at 298.15 K was determined. For some compounds the solubility was shown to be dependent on the pH of the aqueous solution as a result of the protonation processes in the acidic medium. Analysis of the mutual influence of the structural fragments in the molecules on the solubility behavior revealed that the addition of the hydrophilic fragments (substances (4)—glycyl-glycine-memantine and (5)—glycyl-glycyl-glycine-memantine) enhances the solubility, whereas the introduction of the bulky aromatic rings (compounds (**2**)—piracetam-memantine and (**3**)—picamilon-memantine) hampers the dissolution process in both media.

In order to disclose the structure-property correlations in the frame of the studied memantine analogues the physicochemical structural HYBOT descriptors were used. As a result, the significative correlation equations were derived. The equations demonstrated the sensitivity of the solubility in pH 2.0 to the donor ability of atoms in a molecule to form hydrogen bonds (specific interactions) and polarizability (nonspecific interactions), whereas, in pH 7.4 a determinative role of the sum of the acceptor ability to form hydrogen bonds on the solubility was shown. The derived correlation dependences make it possible to predict the solubility of the studied class of compounds based only on their structural formulas.

In the framework of the QSAR approach, the HYBOT descriptors were applied to predict the biological properties (EC_50_) on the class of the memantine derivatives. The best correlation was obtained with the descriptor characterizing the sum of the donor and acceptor ability to form hydrogen bonds normalized to molecular polarizability allowing for both specific and nonspecific interactions of the studied molecules with the biological environment. The equation makes possible the prediction of the EC_50_ values based only on the structural formula, without the expensive biological experiments.

The correlation equations for the solubility and biological properties obtained in this study greatly simplifies the task of the directed design of the memantine analogues with improved solubility and enhanced bioavailability.

The new structural derivatives of memantine were investigated to improve cell viability against copper-induced neurotoxicity in APPswe cells and against glutamate-induced neurotoxicity in SH-SY5Y cells.

Seven novel compounds improved the cell viability of copper-damaged APPswe cells with different effects. Nootropics analogues of memantine (1–3), methyl-glycine (6) and dimethylglycine-memantine (7) are a good EC_50_ value, especially the modafinil-memantine (EC_50_ =1.120 ± 0.398 μΜ). The nootropics analogues of memantine—modafinil-memantine (1) and picamilon-memantine (3) showed cellular cytotoxicity that was similar to the positive drug memantine at 100 μΜ.

Studies against glutamate-induced neurotoxicity in SH-SY5Y cells showed that piracetam-memantine (2) and glycyl-glycine-memantine (4) treated at 0.032 μM significantly improved cell viability of SH-SY5Y cells. These two compounds have a similar neuroprotective effect to the positive drug memantine.

It was revealed that the highest activity combined with low cytotoxicity was demonstrated by the nootropic derivatives of memantine and derivatives of glycine with the simplest in structure—methyl-glycine and dimethylglycine-memantine.

## Figures and Tables

**Table 1:** Solubility of the compounds in buffer solutions pH 2.0 and pH 7.4 at 298.15 K.

№ Compound	pH 2.0	pH 7.4
	S_2_^·^10^3^ (M)	S_2_^·^10^4^ (M)
(**1**)	0.873 ± 0.022	9.93 ± 0.25
(**2**)	1.25 ± 0.03	2.10 ± 0.05
(**3**)	1.02 ± 0.03	1.15 ± 0.03
(**4**)	0.321 ± 0.008	3.44 ± 0.09
(**5**)	6.67 ± 0.17	71.5 ± 1.8
(**6**)	25.88 ± 0.65	201.8 ± 5.1
(**8**) ^a^	205.1 ± 5.1	269.2 ± 6.7
(**9**) ^a^	75.9 ± 1.9	136.1 ± 3.4
(**10**) ^a^	46.2 ± 1.1	83.0 ± 2.1
(**11**) ^a^	118.0 ± 3.0	804 ± 20
(**12**) ^a^	175.0 ± 4.4	164.1 ± 4.1

^a^ solubility was reported in [30]. Compounds numbering corresponds to Figure 2 and Figure 7.

## Data Availability

Data is contained within the article.
